# Supplementary material for: Properties of INDETERMINATE DOMAIN Proteins from Physcomitrium patens: DNA-Binding, Interaction with GRAS Proteins, and Transcriptional Activity
Source: Genes (Basel). 2023 Jun 11;14(6):1249. doi: 10.3390/genes14061249 (PMC10298287; doi:10.3390/genes14061249)
Supplement: Supplementary file 1 [file genes-14-01249-s001.zip › Figure S1.pdf]

Fig. S1

(A)

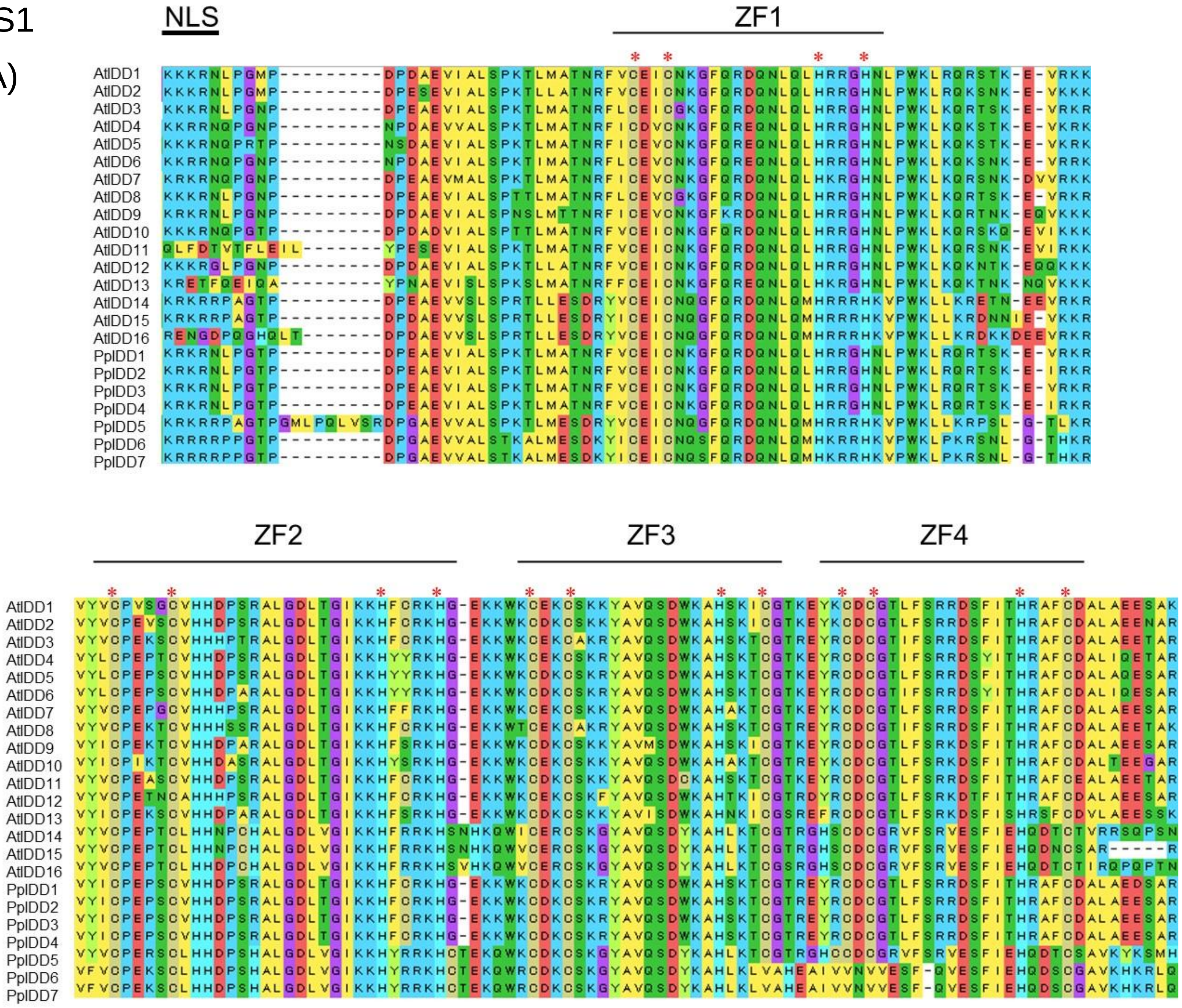
NLS
ZF1
AtIDD1
AtIDD2
AtIDD3
AtIDD4
AtIDD5
AtIDD6
AtIDD7
AtIDD8
AtIDD9
AtIDD10
AtIDD11
AtIDD12
AtIDD13
AtIDD14
AtIDD15
AtIDD16
PpIDD1
PpIDD2
PpIDD3
PpIDD4
PpIDD5
PpIDD6
PpIDD7
ZF2
ZF3
ZF4

Fig. S1

(B)

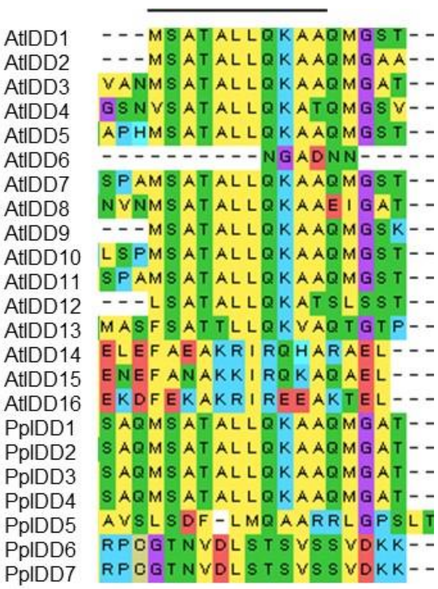
AtIDD1
AtIDD2
AtIDD3
AtIDD4
AtIDD5
AtIDD6
AtIDD7
AtIDD8
AtIDD9
AtIDD10
AtIDD11
AtIDD12
AtIDD13
AtIDD14
AtIDD15
AtIDD16
PpIDD1
PpIDD2
PpIDD3
PpIDD4
PpIDD5
PpIDD6
PpIDD7

(C)

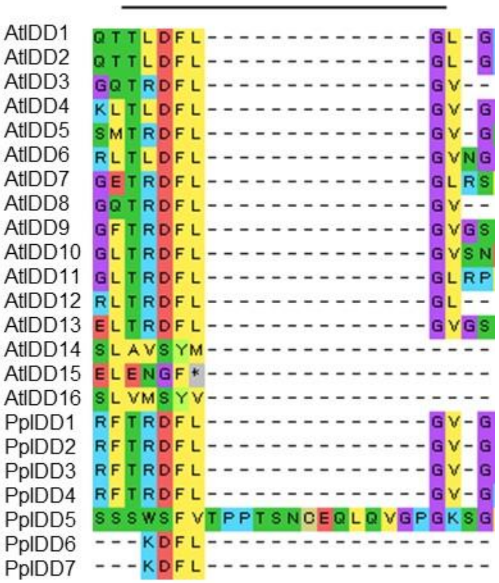
AtIDD1
AtIDD2
AtIDD3
AtIDD4
AtIDD5
AtIDD6
AtIDD7
AtIDD8
AtIDD9
AtIDD10
AtIDD11
AtIDD12
AtIDD13
AtIDD14
AtIDD15
AtIDD16
PpIDD1
PpIDD2
PpIDD3
PpIDD4
PpIDD5
PpIDD6
PpIDD7

Fig. S1

(D)

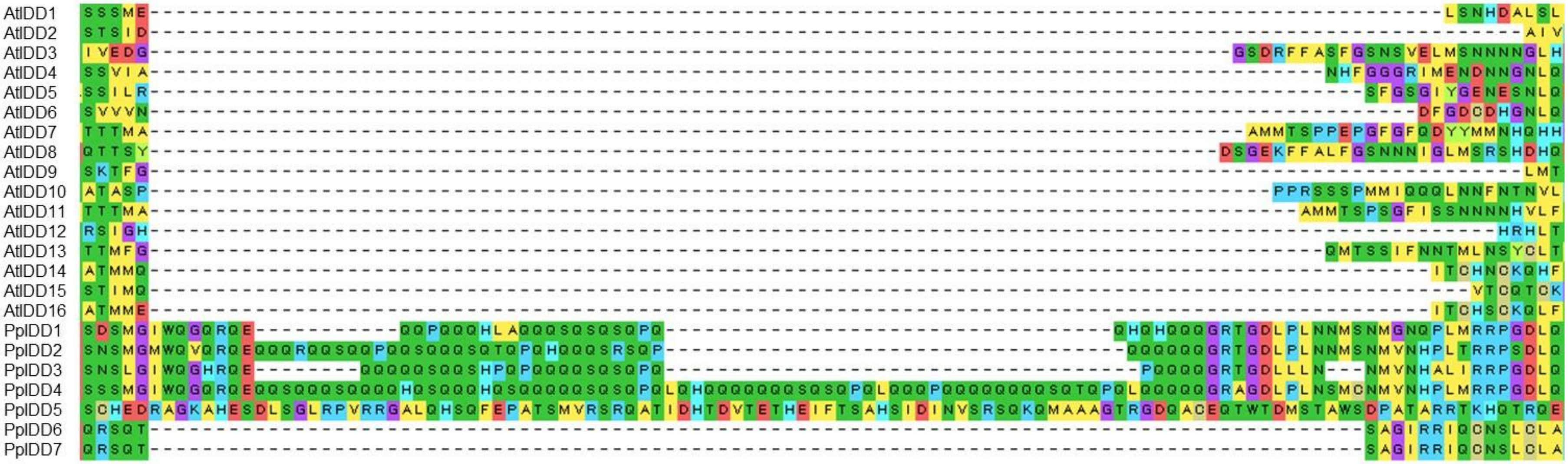

Figure S1

Alignment of amino acid sequences of IDD proteins from *Arabidopsis* and *P. patens*. (A) alignment of IDD domains. The position of each zinc finger (ZF1-ZF4) and NLS are indicated with bar. The position of conserved cysteine (C) and histidine (H) residues are indicated with asterisks. The sequences around (B) the MSATALLQKAA motif and (C) the T[R/L]DFLG motif in the C-terminal regions. Black bars indicate the conserved sequences. (D) Q-rich sequences in the C-terminal regions of PpIDD1-4. The sequence alignment was made using MEGA-11 and ClustalW. The colors indicate amino acids of different biochemical properties as obtained through MEGA 11.
