# Supplementary material for: Properties of INDETERMINATE DOMAIN Proteins from Physcomitrium patens: DNA-Binding, Interaction with GRAS Proteins, and Transcriptional Activity
Source: Genes (Basel). 2023 Jun 11;14(6):1249. doi: 10.3390/genes14061249 (PMC10298287; doi:10.3390/genes14061249)
Supplement: Supplementary file 1 [file genes-14-01249-s001.zip › Table S1.pdf]

Table S1. Primers used in this study

primers for yeast experiments

gene	vectors		sequence
PpIDD1	pGAD424, pGBT9	F	5´-GGAATTCATGACATTGT CCAACTTAACC-3´
		R	5´-GGGATCCTAGGTGGAGGGG TCCCATGACT TCC-3´
PpIDD2	pGAD424	F	5´-GGTCGACCTATGACATTGT CCAACTTAACC-3´
		R	5´-GAGATCTCTAG TTGGCGTCCC ATGACTTTCC-3´
	pGBT9	F	5´-GGATATCGATGACATTGTCCAACTTAACC-3´
		R	5´-GGTCGAC CTAG TTGGCGTCCC ATGACTTTCC-3´
PpIDD3	pGAD424, pGBT9	F	5´-GGATATCGATGACATTGT CCAACTTAACC-3´
		R	5´-GGTCGACCTAGGTGGCGTC CCATGACTTCC-3´
PpIDD4	pGAD424	F	5´-GGTCGACCTATGACATTGT CAAATTTAACC-3´
		R	5´-GAGATCTCTAGGTAGCG GCGTCCCATG-3´
	pGBT9	F	5´-CGGATATCGATGACATTGTCAAATTTAACC-3´
		R	5´-GGTCGAC CTAGGTAGCG GCGTCCCATG-3´
PpIDD5	pGAD424, pGBT9	F	5´-GGAATTCATGAACGCCT TACCTTCAGC-3´
		R	5´-G GTCGACTCAGTTC AAATCGAAAT CCTTTGG-3´
PpDELLAa	pGAD424, pGBT9	F	5´- GGGATCC GTATGGCGTATC AGTACTCTCC-3´
		R	5´- GGTCGAC TCATGCACAT TTCCATGCAG-3´
PpDELLAb	pGAD424, pGBT9	F	5´-GGTCGAC CTATGGCATATC AGTACTATCC-3´
		R	5´-GCTGCAG TCATGCACAT TGCCATGCAG-3´
PpSHR 2	pGAD424, pGBT9	F	5´-GGGATCC GTATGGACAAAC TTCTGGTAAA ATATC-3´
		R	5´-GGTCGAC TCATGGTTCC GGTGACGGGC-3´

primers for transient assay

gene	vectors		sequence
GBD F	35S:pUC19	F	5' -CCTGCAGGTCGACTCTAGAGATGAAGCTACTGTCTTCTATC- 3'
PpIDD2	35S:pUC19	F	5' -CCTGCAGGTCGACTCTAGAGATGACATTGTCCAACTTAACC- 3'
		R	5' -GAGCTCGGTACCCGGGGATCCTAGTTGGCGTCCCATGACTT- 3'
PpIDD4	35S:pUC19	F	5´-G TCTAGA ATGACATTGT CAAATTTAACC-3´
		R	5´-GAGATCTCTAGGTAGCG GCGTCCCATG-3´
PpIDD5	35S:pUC19	F	5' -CCTGCAGGTCGACTCTAGAGATGAACGCCTTACCTTCAGCT- 3'
		R	5' -GAGCTCGGTACCCGGGGATCTCAGTTCAAATCGAAATCCTT- 3'
PpDELLAa	35S:pUC19	F	5´-G TCTAGA ATGGCGTATC AGTACTCTCC-3'
		R	5´-GGGATCC TCATGCACAT TTCCATGCAG-3´
PpDELLAb	35S:pUC19	F	5´-GTCTAGA ATGGCATATC AGTACTATCC
		R	5´-G AGATCT TCATGCACAT TGCCATGCAG
PpSHR 2	35S:pUC19	F	5´-G TCTAGA ATGGACAAAC TTCTGGTAAA ATATC
		R	5´-GGGATCC TCATGGTTCC GGTGACGGGC
